# Evolution of Catkins: Inflorescence Morphology of Selected Salicaceae in an Evolutionary and Developmental Context

**DOI:** 10.3389/fpls.2015.01030

**Published:** 2015-12-07

**Authors:** Quentin C. B. Cronk, Isabelle Needham, Paula J. Rudall

**Affiliations:** ^1^Department of Botany, University of British ColumbiaVancouver, BC, Canada; ^2^Royal Botanic Gardens, Kew, LondonUK

**Keywords:** inflorescence evolution, heterochrony, synorganization, preformation, dioecy, floral reduction, inflorescence architecture, genome-enabled model system

## Abstract

Poplars (*Populus* sp.) and willows (*Salix* sp.) are well known woody plants common throughout the northern hemisphere, both with fully sequenced genomes. They bear compact unisexual inflorescences known as “catkins.” Closely related genera of the “salicoid clade” within the family Salicaceae include the Asian genera *Bennettiodendron*, *Idesia*, *Itoa*, *Poliothyrsis*, and *Carrierea* and the Central American genera *Olmediella* and *Macrohasseltia*. Like willow and poplar, most of these genera are dioecious, but unlike willow and poplar they generally have loosely branched panicles rather than catkins, and less highly reduced flowers. However, the early developing inflorescences of *Carrierea* and *Idesia* show similarities to catkins which suggest possible pathways by which the salicoid catkin may have evolved.

## Introduction

### The Catkin and its Recurrent Evolution

The catkin is a type of compact or string-like inflorescence characterized by a single relatively stout axis on which unisexual sessile or subsessile apetalous flowers are clustered in a spiral or whorled arrangement. It is an extremely striking characteristic of many common trees, particularly of northern temperate regions. Notable among these are members of the order Fagales (oaks, walnuts, hazels, birches, and alders) and the relatively distantly related family Salicaceae s. str. (willows and poplars). The similarities between the catkins of these two groups led to them being classified together for a century (see below). It is now accepted that the presence of catkins in the two groups is the result of convergent evolution.

In this paper ‘catkin’ will be used in preference to the alternative term ‘ament.’ According to the Oxford English Dictionary, the word catkin came into English in 1578 when Henry Lyte (1529-1607) coined it in his translation of Dodoens’ New Herbal as a translation of the Dutch “katteken” (kitten) used for the downy inflorescences of willows and other plants (Dodoens, 1578). The botanical Latin equivalent, *amentum*, the Latin word for a thong or string, is less common. Its use in English dates from the late 18th century, sometimes anglicized as ament. The use of *amentum* in botanical Latin overlooks the Latin word for catkin, *iulus*, used as such by Pliny. However, apart from the occasional use of Juliflorae instead of Amentiflorae, this form never became established.

### The Catkin and Taxonomy

**FIGURE 1:**
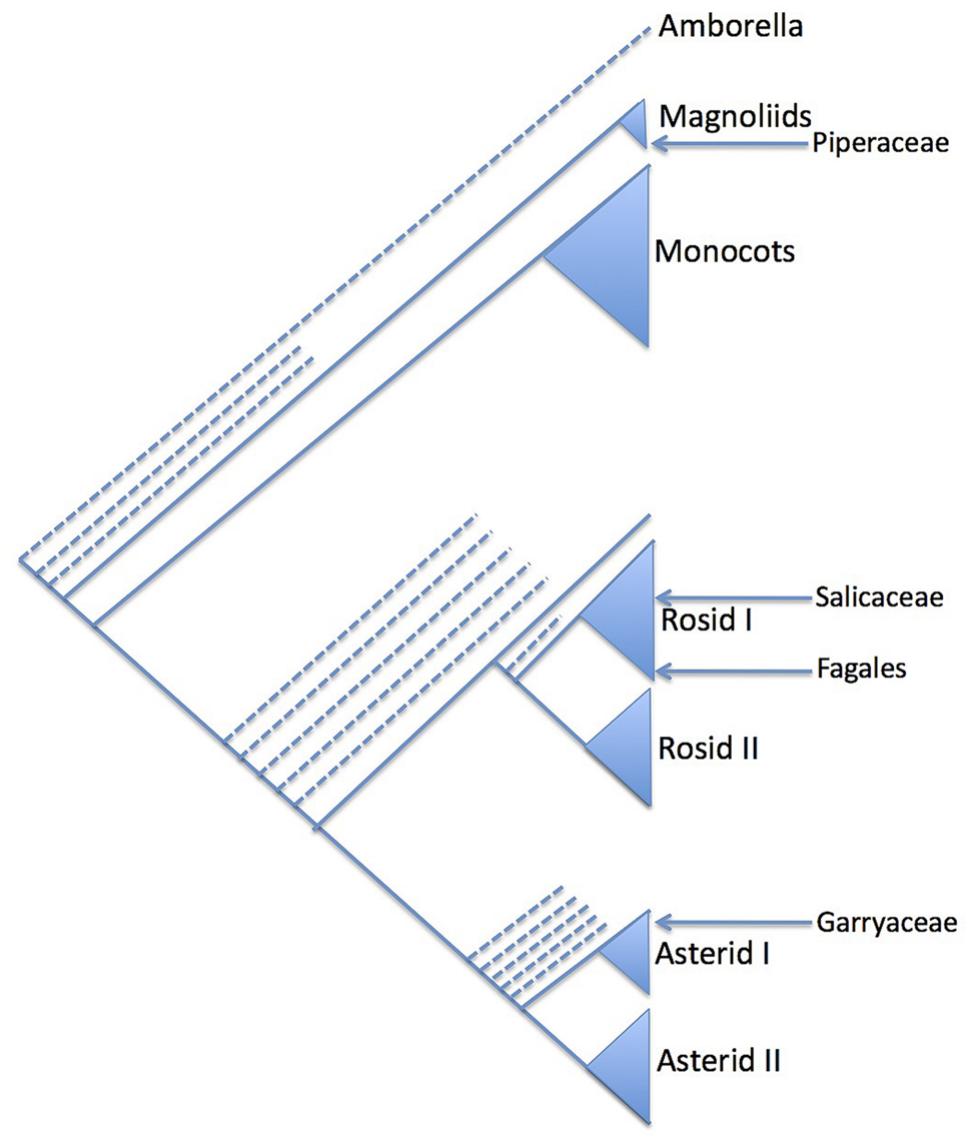
**Summary phylogenetic diagram showing the relative phylogenetic position (arrowed) of selected groups that have formerly been included in the Amentiferae due to their catkins or catkin-like inflorescences (see text)**. The phylogeny reflects that of the Angiosperm Phylogeny Group (2009). Only major clades are shown: the position of minor clades is indicated with dashed lines.

The striking amentaceous inflorescences of many trees quickly attracted the attention of botanists, some of whom thought that the catkin-bearing trees formed a natural group (variously called Amentiflorae, Amentiferae, Amentales, or Amentaceae; Stern, 1973). Although the group name “Amentacea” was used by Gmelin, Linnaeus, and de Jussieu (Stern, 1973) and sporadically by later authors (Du Mortier, 1825), it was Eichler who was most influential in defining a ‘scientific’ Amentaceae. In the third edition of Eichler’s Syllabus (Eichler, 1883) the order Amentaceae comprised the Cupuliferae (i.e., Betulaceae and Fagaceae s.l.), Juglandaceae, Myricaceae, Salicaceae, and Casuarinaceae. This collection of families can be considered the canonical Amentiferae, although other groups have drifted in and out of the catkin-bearing alliance in various systems, e.g., Piperaceae, Urticales, *Leitneria*, Garryales (**Figure 1**). Remarkably, Eichler’s (1883) Amentaceae is a good natural group (providing of course that the unrelated Salicaceae is excised). In fact it corresponds almost exactly to the modern concept of the Fagales (Angiosperm Phylogeny Group, 2009), missing only *Rhoiptelea* and *Ticodendron*, both unknown to Eichler.

In the last century the realization grew that the Salicalean branch of the Amentiferae was very different from the Fagalean Amentiferae (**Figure 1**). This realization was confirmed by molecular phylogenetics and made clear in systems based on molecular phylogenetics such as the Angiosperm Phylogeny Group (APG) system (Angiosperm Phylogeny Group, 2009). An implication of the decomposition of the Amentiferae is that catkins, the most obvious unifying feature of the Amentiferae, have evolved in two very distinct lineages. This raises questions of convergent evolution: how the catkin evolved in each case and what the ancestral inflorescence form might be. Here we use comparative ontogenetic and anatomical observations as a basis to discuss these questions in one of the archetypal catkin-bearing groups, Salicaceae.

### The Salicaceae, Classification and Morphology

When Eichler included Salicaceae within his order Amentaceae, the family was wholly amentiferous (i.e., catkin-bearing) comprising only the genera *Salix* and *Populus*. Molecular evidence, coupled with support from phytochemistry and morphology, has demonstrated a close relationship between *Salix* and *Populus* and many non-amentiferous genera that were formerly placed in the Flacourtiaceae (Leskinen and Alstrom-Rapaport, 1999; Chase et al., 2002; Alford, 2005). The heterogeneous family Flacourtiaceae is now dismembered, and its members are placed in other families, mainly the Salicaceae and Achariaceae. The family Salicaceae, as now circumscribed in the broad sense, is a more homogeneous group of about 1000 species in c. 55 genera. They are uniformly woody (trees or shrubs) with simple, usually alternate, leaves. The leaves are often dentate and the leaf teeth frequently glandular (characteristic ‘salicoid teeth’). The flowers are often inconspicuous and a perianth may be lacking in some genera. Inflorescence morphology in the family as a whole is highly variable. The sister family to the Salicaceae is probably the Lacistemataceae (Davis et al., 2005; Korotkova et al., 2009), and it is of interest that this family has also independently evolved catkins.

**FIGURE 2:**
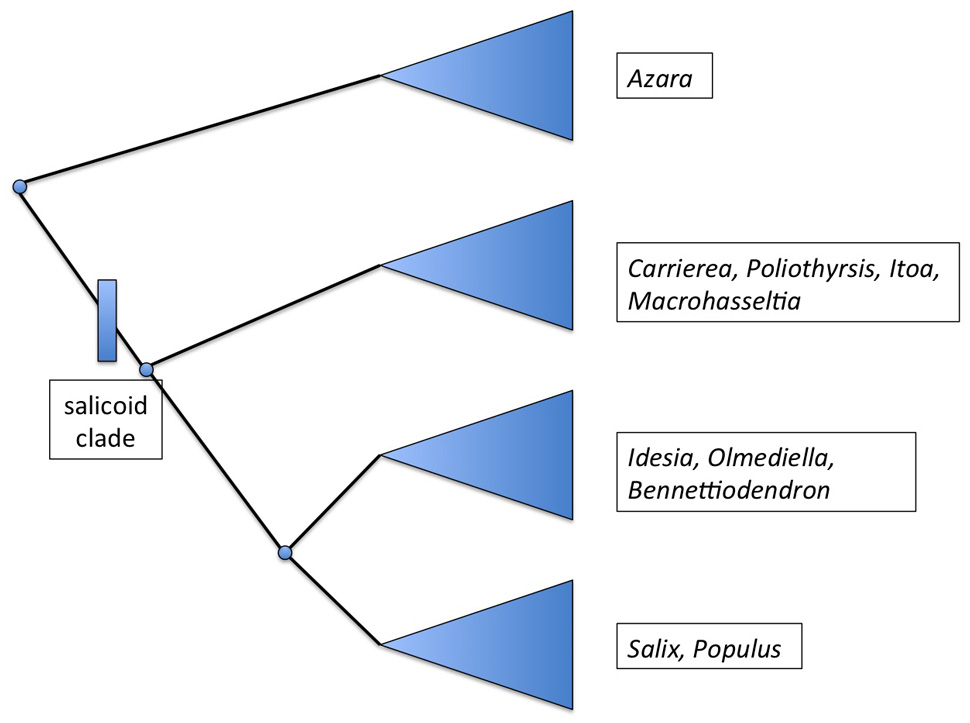
**Summary phylogenetic diagram showing the major groupings within the salicoid clade, after Alford et al. (2009)**.

*Salix* and *Populus* are closely related sister genera which in turn are related to a group of seven other genera (Alford, 2005). Initial molecular phylogenetic evidence based on ITS and eight plastid regions suggests that “a clade consisting of *Bennettiodendron*, *Idesia*, and *Olmediella* are sister to *Salix* and *Populus* (**Figure 2**). Sister to that clade is a clade of the other four genera, *Carrierea*, *Itoa*, *Macrohasseltia*, and *Poliothyrsis*” (Alford et al., 2009).

These nine genera have been referred to as the “salicoid clade” of the family Salicaceae (Cronk, 2005). *Salix* and *Populus* are known to be palaeotetraploid (Sterck et al., 2005) with a primary chromosome number of *n* = 19 (Darlington and Wylie, 1955) whereas the haploid base number for the family is *n* = 9 or 11. For instance, *Azara serrata* Hook is *n* = 9 (Sanders et al., 1983). Very few chromosome counts exist for the genera of the salicoid clade but both *Olmediella* (Grill, 1990) and *Idesia* (Darlington and Wylie, 1955; Grill, 1990) appear to be tetraploid at *n* = 22. It is possible therefore that whole of the salicoid clade shares the same palaeotetraploidy event from *n* = 11 to *n* = 22, followed by (in *Populus* and *Salix*) reduction events to *n* = 19.

### The Inflorescence of the Salicoid Clade of the Salicaceae

**FIGURE 3:**
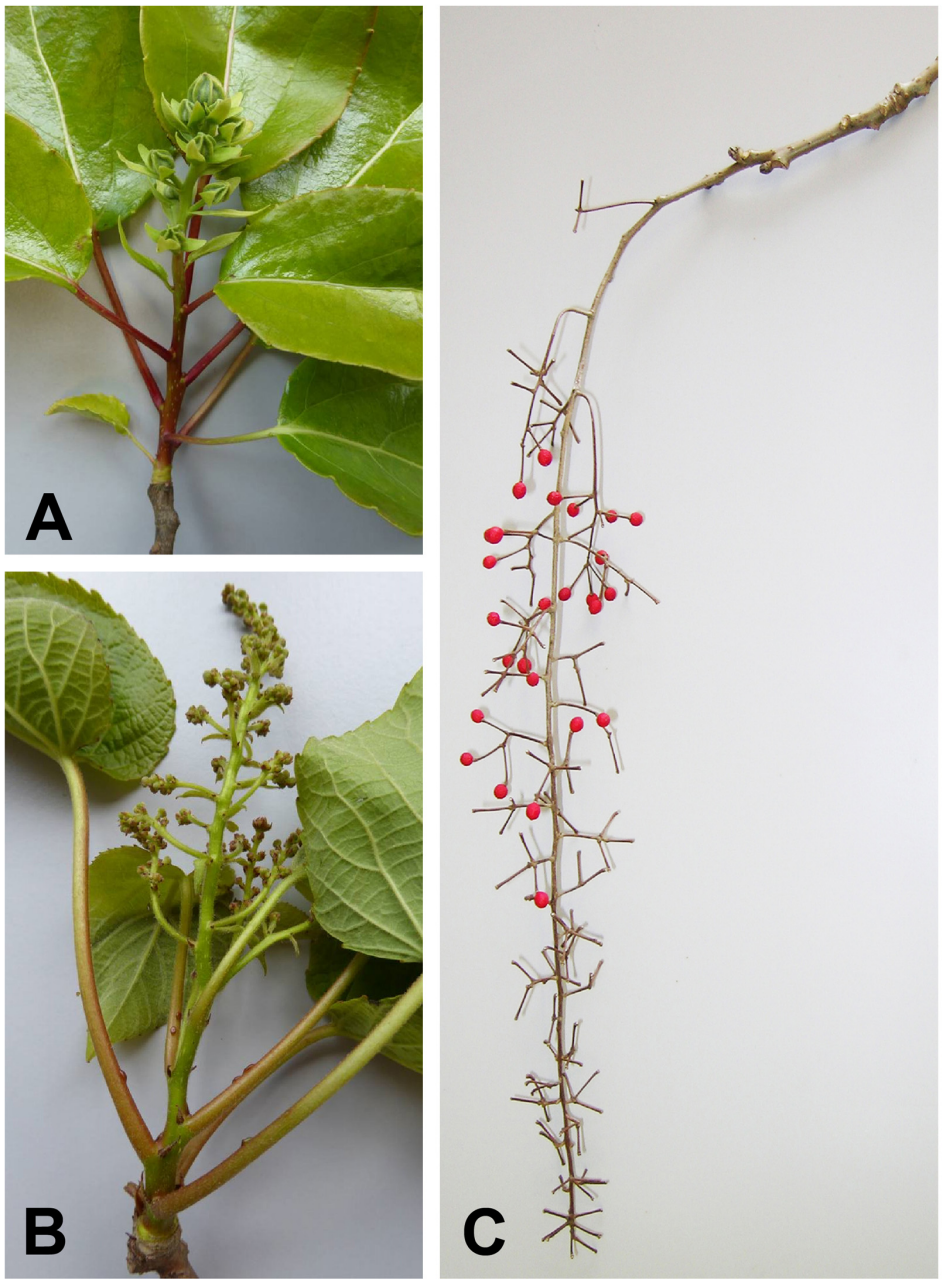
**Photographs of inflorescences. (A)** Young inflorescence of *Carrierea calycina* Franch.) collected May 2015. The flowers are unopened. The floral bracts and calyces are visible. **(B)** Young male inflorescence of *Idesia polycarpa* Maxim., collected May 2015. The flower buds are still small but the branching of the paniculate inflorescence is evident. This inflorescence has developed in 3 months from an inflorescence meristem as shown in **Figure 5B. (C)** Previous year’s female inflorescence (infructescence) of *I. polycarpa*, collected February 2015, showing the extensive branching of the inflorescence.

The genera of the salicoid clade have been described in various recent treatments (Sleumer, 1980; Fang et al., 1999; Yang and Zmartzty, 2007). They are all described as having paniculate inflorescences with the exception of *Populus* and *Salix* which have racemose inflorescences (catkins). A reduction of inflorescence branching (from panicle to raceme) does occur as a sexual dimorphism in *Itoa*, in which the male flowers are said to be in racemes and the female flowers in panicles (**Table 1**). Furthermore, in *Idesia* the paniculate inflorescences are long, pendulous and fairly narrow, superficially resembling racemes (**Figure 3**).

**Table 1:** Genera of the salicoid clade of Salicaceae.

Genus	No. of sp.	Sex of flowers	Distribution of sexes	Inflorescence	Perianth and disk
*Bennettio-dendron*	3 (Asia)	Unisexual	Dioecious	Terminal or axillary panicles	Sepals 3, petals 0, disk glands numerous, small
*Carrierea*	2 (Asia)	Unisexual	Dioecious	Short terminal or axillary panicles	Sepals 5, petals 0, disk glands 0
*Idesia*	1 (Asia)	Unisexual	Dioecious	Long raceme-like terminal or axillary panicles	Sepals c.5, petals 0, disk glands numerous among stamens or staminodes
*Itoa*	1 (Asia)	Unisexual	Dioecious (or partly monoecious?)	Terminal panicles (f) or terminal or axillary racemes (m)	Sepals 5, petals 0, disk glands 0
*Macrohasseltia*	1 (S. Am.)				
*Olmediella*	1 (C. Am.)	Unisexual	Dioecious	Small panicles	Sepals 5 (reduced), petals 0, disk glands numerous (at base of each stamen)
*Poliothyrsis*	1 (Asia)	Unisexual	Monoecious	Terminal panicle, upper flowers female	Sepals 5, petals 0, disk glands 0
*Populus*	c. 60 (wide)	Unisexual	Dioecious	Catkin	Sepals 0, petals 0, disk cupular
*Salix* (incl. *Chosenia* which has both disk glands absent)	c. 500 (wide)	Unisexual	Dioecious	Catkin	Sepals 0, petals 0, disk glands us. 2 (adaxial and abaxial; abaxial may be absent)

In the fossil record there is some blurring between raceme and panicle. *Pseudosalix*^†^ (Boucher et al., 2003) is an Eocene fossil of Salicaceae that has leaves like willow (*Salix*) but somewhat paniculate inflorescences. Furthermore, the Eocene *Populus tidwellii*^†^ Manchester, Judd & Handley (Boucher et al., 2003; Manchester et al., 2006) has catkins with some lateral branching near the base, placing it in an intermediate position in this character with the paniculate ancestors of *Populus*. Furthermore, Fisher (Fisher, 1928a,b) argued that the bract (with which the flower is associated), while appearing to be directly inserted on the main axis of the inflorescence, is in fact inserted on a minute lateral stump (which Fisher called the “internode”) at the top of which the flower is borne. She argued that this feature is an indication of the evolution of the Salicaceae catkin from a branched, paniculate antecedent.

In most of the salicoid clade, the inflorescence is terminal on the shoots, often (as in *Idesia*, *Carrierea*, and *Poliothyrsis*) terminating the shoots that appear after bud-break of the terminal bud. This condition contrasts with *Salix* and *Populus*, in which the inflorescences are produced in lateral buds (with very few exceptions: in *Sali*x sect. *Chamaetia* they are often terminal). Again, *P. tidwellii*^†^ (Manchester et al., 2006) is interesting in this regard as it has terminal inflorescences.

### Floral Morphology of the Salicoid Clade

Apart from unisexuality, lack of petals and the presence of nectarial disk glands in some species, the flowers of the most genera in the salicoid clade are unexceptional.

The flowers all have a subtending bract. The highly reduced flowers in *Salix* and *Populus* prompted some early authors to suggest that the bracts in thes taxa might be derived from the missing perianth. However, this interpretation was shown to be false by Fisher (1928a,b) who demonstrated that they have a foliar-type vascularization consistent with bract origin: these bracts are therefore simply homologous with the floral bracts of other members of the salicoid clade.

All genera have a calyx of (3-)5(-6) sepals, except *Salix* and *Populus* which lack an obvious perianth entirely. It should be noted that in *Olmediella* the calyx is reduced and quickly caducous (Alford, 2005). The staminate flowers have numerous stamens (in all except *Salix*, which generally has only 1–5 stamens) and a vestigial ovary (absent in *Salix* and *Populus*). The pistillate flowers have numerous small staminodes (except *Populus* and *Salix*). The presence of vestigial sexual organs in most species but their complete absence in *Salix* and *Populus* indicates the extent of the process of floral reduction in those genera. However, it is not known whether the developmental pathways for vestigial pistils in males (and staminodes in females) has been completely removed as part of sex determination or merely reduced to the extent it no longer has anatomical consequence.

The nectarial disk glands are an important floral feature in many genera (**Table 1**). These glands are generally assumed to be outgrowths of the disk. However, they are consistently associated with the stamens and staminodes, appearing interspersed among the stamens (intrastaminal, as in *Idesia*), or at the bases of the stamens in *Olmediella* (Alford, 2005). This location raises the question of whether they might be staminodial in origin.

There is a further question of whether the disk glands (nectaries) of *Salix* are homologous with the disk glands of other genera. Fisher has argued convincingly (Fisher, 1928a,b) that they represent a modified perianth because there appears to be some vascularization. However, Fisher made this argument before the outgroups of *Salix* were known. Now that we know the close relationship between *Salix* and other genera with disk glands, it seems logical to assume their homology (Alford, 2005).

Another puzzle is the “cupular disk” of *Populus*. Fisher homologised this structure with perianth and with the disk glands of *Salix*: “The disk-shaped perianth of *Populus*, or its peripheral parts, is homologous with the nectary of *Salix*” (Fisher, 1928b). Skvortsov (1999) also had no difficulty homologizing the disk glands of *Salix* with the cupular disk of *Populus*, mainly because the disk glands in *Salix* are sometimes united and approach in morphology the cupular disk of *Populus*. He writes: [*Salix* has]“...one or two (or a few) nectariferous glands, which occasionally are connate into a lobed glandular disk. These glands are obviously homologous to the cup-shaped disk in the poplars (which is sometimes called perianth).” However, the cupular disk of *Populus* is vascularized, consistent with Fisher’s thesis that the disk glands of *Salix* have a perianth origin, assuming the two to have a common origin. However, it is also possible that as non-perianth disk glands evolved to increased complexity, vascularization was co-opted. Another possibility is that the cupular disk of *Populus* is indeed directly homologous with the calyx but not with the glands of *Salix* (which then have a non-perianth origin). Finally, the simplest explanation of all is that the cupular disk is merely an enlarged disk (i.e., receptacular in origin).

In this paper we seek to investigate whether the morphology of closely related non-catkin-bearing species can inform our understanding of the evolution of catkins in the Salicaceae. In particular we are interested in setting out the main ways in which *Salix* and *Populus* differ, in reproductive morphology and phenology, from their close relatives. Knowledge of the inflorescence morphology and flowering behavior of related plants allows the formulation of scenarios by which catkins evolved in this clade.

## Materials and Methods

### Sample Collection

For *Idesia*, *Carrierea*, and *Poliothyrsis*, terminal resting buds were collected in spring before bud-break. In *Salix* and *Populus* lateral inflorescence buds were collected at the same time. A young inflorescence of *Olmediella* was collected at the same time from greenhouse-grown material. After budbreak young inflorescence shoots were also examined. A list of samples collected with accession numbers is given in **Table 2**.

**Table 2:** Details of material examined in this study.

Name	RBG Kew accession no.	Date collected
*Carrierea calycina* Franch. –F	2006-616	6.2.2015, 13.2.2015
*Idesia polycarpa* Maxim. – F	2006-332	6.2.2015, 13.2.2015
*Idesia polycarpa* – M	2008-416	6.2.2015, 13.2.2015
*^∗^Olmediella betschleriana* (Göpp.) Loes. – F	1969-12335	16.2.2015
*^∗^Poliothyrsis sinensis* Oliv. – Mon	1973-20904	7.2.2015, 13.2.2015
*Populus nigra* L. – M	1988-8331	2.2.2015
*Populus purdomii* Rehder – F	1973-6401	2.2.2015
*Populus wilsonii* C.K. Schneid. – F	1979-1110	18.2.2015
*Salix miyabeana* Seem. – M	1999 - 547	16.2.2015
*Salix* sp. – F	1999-548	16.2.2015

*All material was collected from living material growing in the Royal Botanic Gardens Kew. Male and female plants are indicated as M and F, respectively (Mon, monoecious). An asterisk indicates those species not figured in the present study*.

### Sample Preparation

Collections of inflorescence material were killed and fixed in formalin–acetic acid–alcohol (FAA) for approximately 1 week followed by storage in 70% ethanol. Some material was dehydrated through an ethanol series to 100% ethanol, transferred to Histoclear before embedding in Paraplast using standard protocols. The wax blocks were sectioned at 10 μm thickness on a rotary microtome (Leica RM2155) and the resulting sections were stained in 0.5% (w/v) solution of toluidine blue before mounting on microscope slides in DPX mountant. Images were captured using a Zeiss Axiocam HRc camera attached to a Leica DMLB microscope.

Other material was dehydrated through an alcohol series into acetone and transferred to a critical-point drier (Tousimis Autosamdri 815B). Dried material was then sputter-coated with platinum in a sputter coater (Emitech K550). The material was examined on a Hitachi S-4700 II cold-field emission scanning electron microscope.

## Results

### Phenology and Gross Morphology of Reproductive Shoots

In autumn *Poliothyrsis*, *Idesia*, and *Carrierea* set comparatively large terminal buds on all shoots of the previous year. The majority of these buds produce short shoots terminating in an inflorescence (**Figure 3**). Vegetative growth (and flowers of the following year) is therefore left to side shoots. This growth pattern corresponds with the “Modèle de Leeuwenberg” of Hallé and Oldeman (Hallé et al., 1978). *Populus* has the opposite tendency, with terminal buds tending to be vegetative and side shoots (from axillary buds of the previous year) tending to contain catkins. In *Poliothyrsis*, nearly all growth is by terminal buds from side shoots of the previous year, these in turn terminate in inflorescences with between four and six leaves below then. In *Carrierea* there tend to be four to six leaves below each inflorescence and in *Idesia* four to five.

### Developmental Anatomy

**FIGURE 4:**
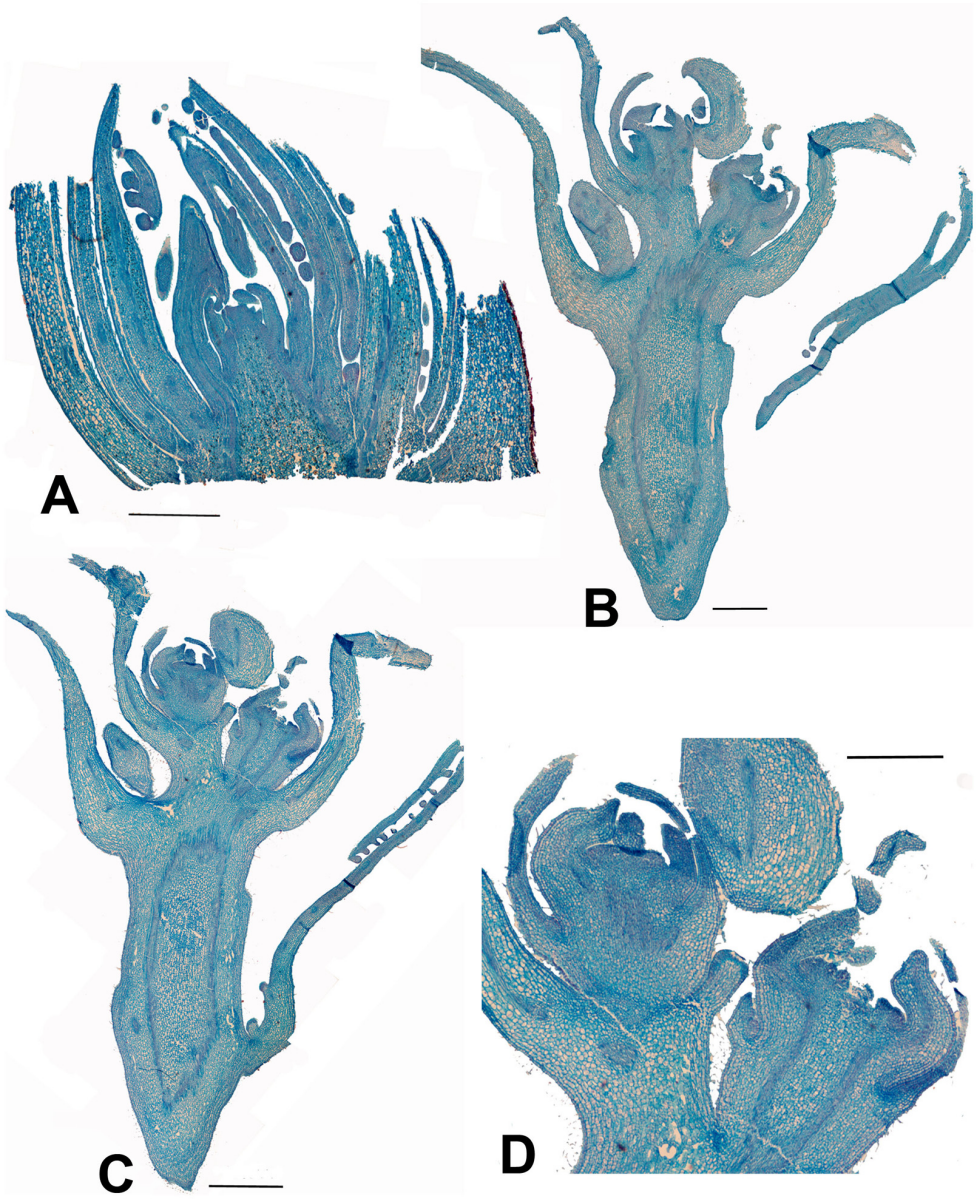
***Carrierea calycina*. (A)** Over-wintering bud with minute inflorescence meristem surrounded by protective bracts (bud scales) collected 13th February 2015. At this stage the inflorescence has not started developing. **(B,C)** Sequential longitudinal sections of dissected resting bud (pre-budbreak) collected 2nd April 2015, showing a fairly well-developed inflorescence with individual flowers differentiated. Floral bracts and calyx are visible but other organs have not formed and gender is not visible at this stage (bud scales removed). **(D)** Detail of developing flower from **(C)**. Scale bars = 1 mm.

**FIGURE 5:**
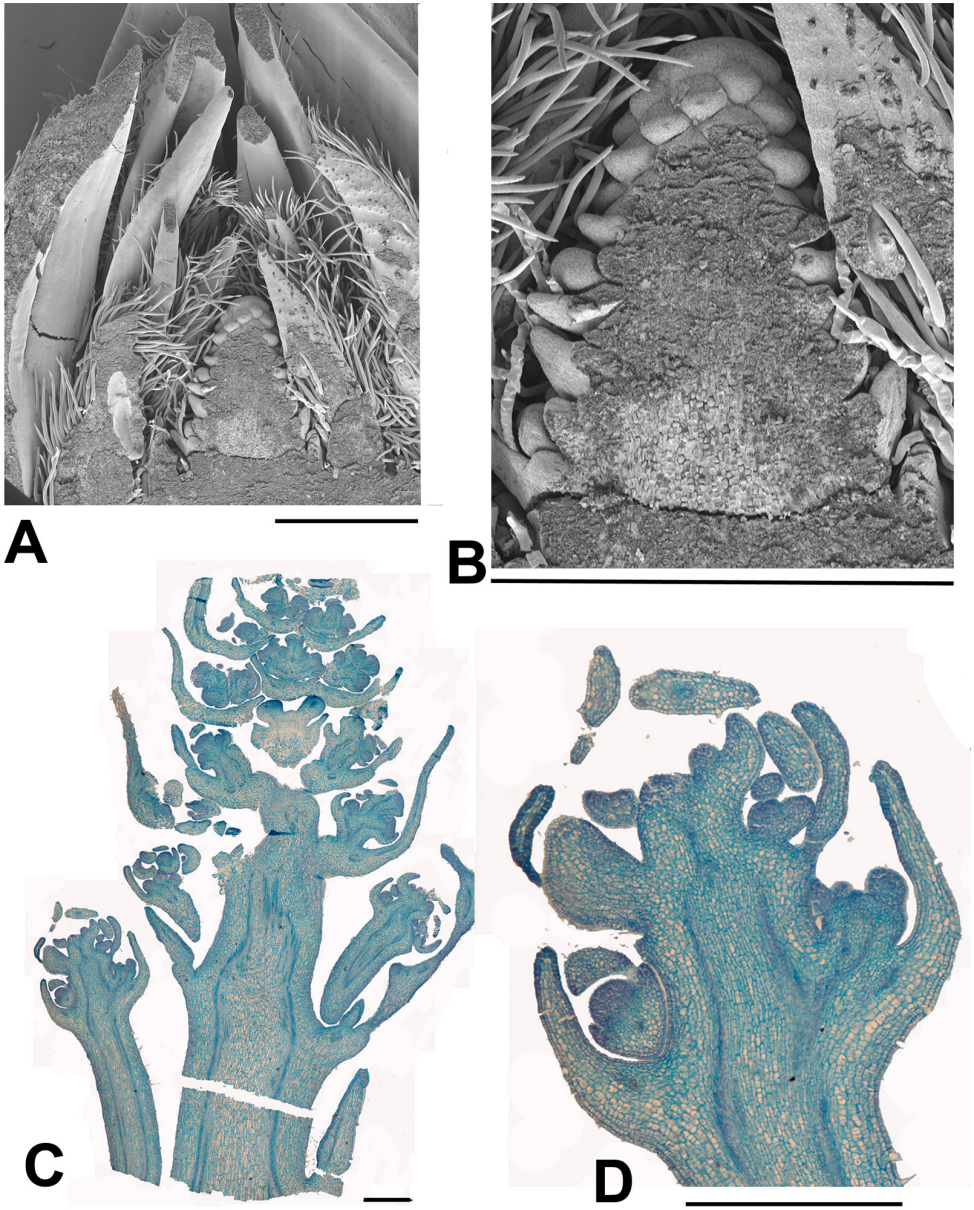
***Idesia polycarpa*. (A)** SEM of bisected over-wintering bud collected 13th February 2015 showing an inflorescence meristem surrounded by protective bracts (bud scales; **B**) Detail of **(A)** showing just the inflorescence meristem, At this stage the inflorescence meristem shows inflorescence bract primordia but not floral primordia. Later branch primordia will form in the axils of the inflorescence bracts and develop into the paniculate (i.e., branched) inflorescence. At the same stage *Salix* and *Populus* inflorescences are fully developed with completely formed flowers (**Figures 6** and **7**). **(C)** Longitudinal section of dissected over-wintering (pre-budbreak) bud collected 2nd April 2015 showing development of panicle (bud scales removed). Flowers can be seen in early development, with some development of the calyx but not other organs. At the same date *Salix* and *Populus* have finished flowering. **(D)** Enlarged portion of **(C)**. Scale bars = 1 mm.

**FIGURE 6:**
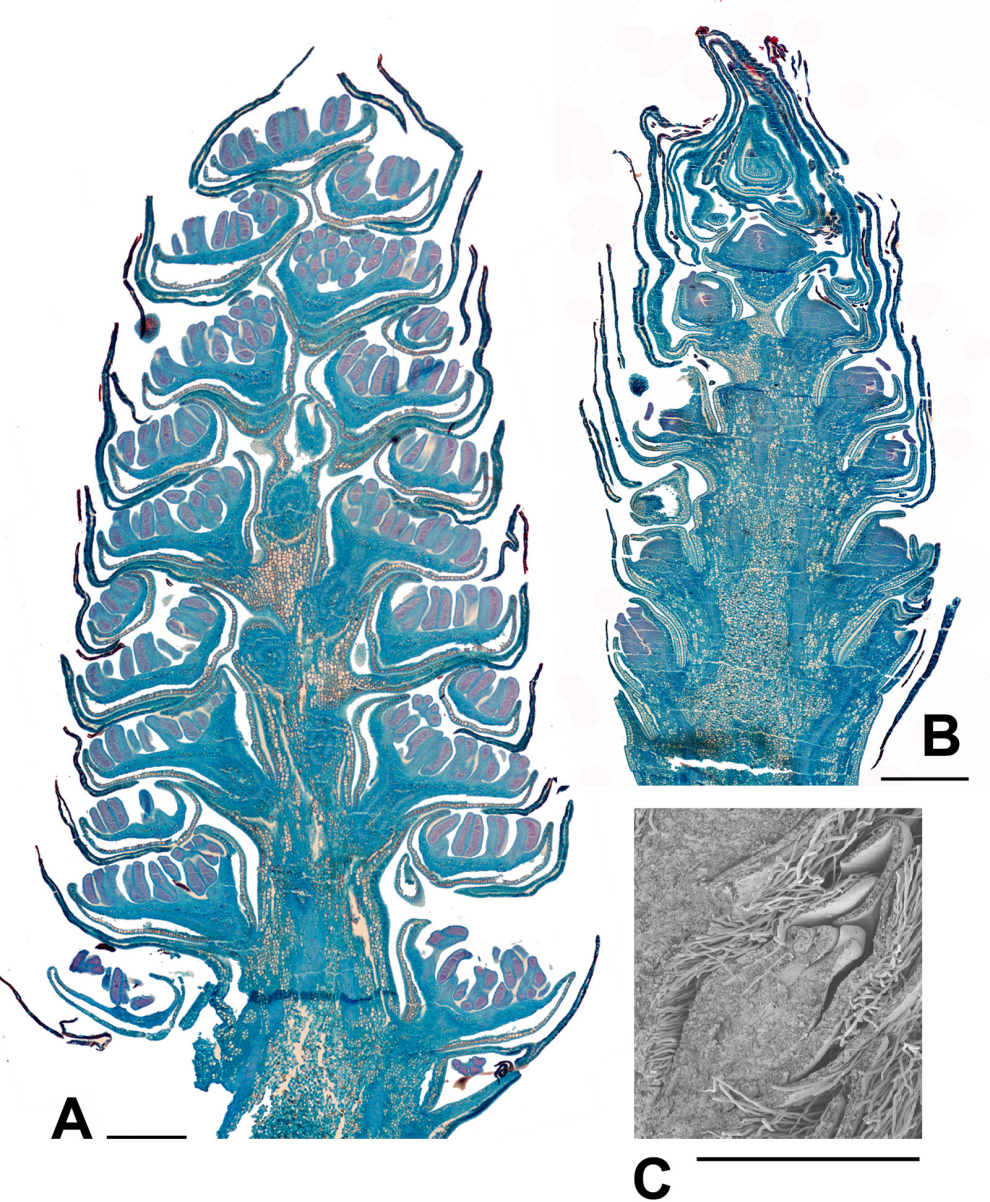
***Populus*. (A)** *Populus nigra* L., male inflorescence (catkin) with nearly fully developed flowers. Longitudinal section of catkin, collected 2nd February 2015, showing well-developed stamens with short filaments and longer anthers with red-staining developing pollen (bud scales removed). **(B)** *P. purdomii* Rehder, female inflorescence with flowers in a late stage of development. Longitudinal section of catkin collected 2nd February 2015, showing single ovary per flower. **(C)** *P. wilsonii* C.K.Schneid., SEM detail showing a well developed ovary from a female inflorescence, collected 2nd February 2015. Scale bars = 1 mm. Bud scales removed.

**FIGURE 7:**
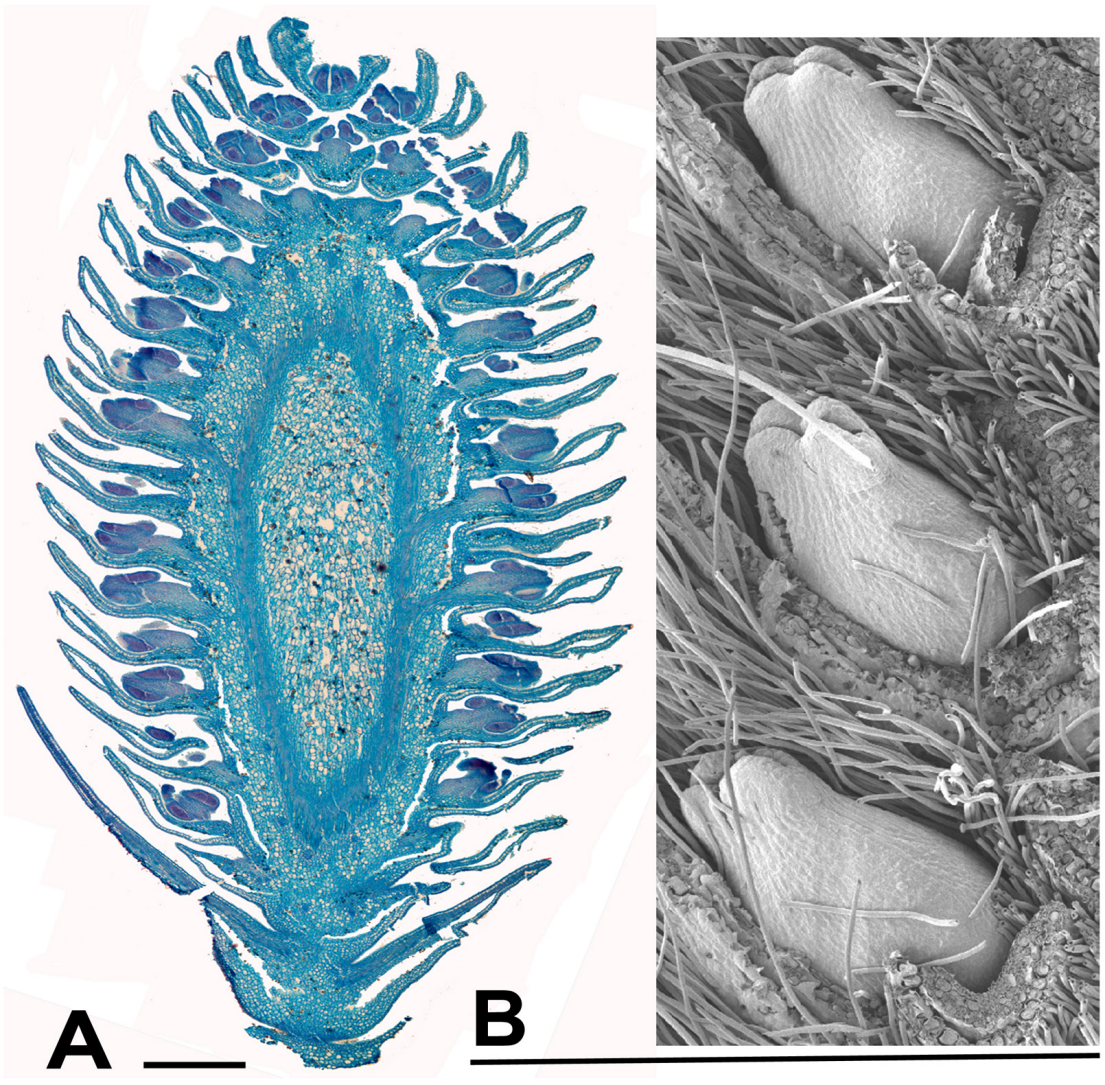
***Salix*. (A)** *Salix miyabeana* Seem., male inflorescence (catkin). Longitudinal section of over-wintering bud (bud scales removed), collected 16th February 2015, showing well-developed flowers, each with an apparent single stamen (actually two stamens completely united except at the anther). The anthers are well differentiated pre-anthesis. **(B)** *Salix* sp., SEM showing three well developed female flowers in the axils of bracts, from an inflorescence in over-wintering bud collected 16th February 2015. Scale bars = 0.5 mm. Bud scales removed.

*Carrierea* (**Figures 4A–D**) and *Idesia* (**Figures 5A,B**) show very little development of the inflorescence when collected in February. In contrast, *Populus* and *Salix* (**Figures 6** and **7**) have fully formed flowers. The preformation and early development of inflorescences in *Populus* and *Salix* is well known, with inflorescences formed the previous year (Boes and Strauss, 1994; Kaul, 1995; Brunner et al., 2014). **Figures 6** and **7** show the almost fully developed flowers inside the unopened buds enclosing catkins of *Populus* and *Salix* when sampled in early February (well before bud opening and flowering in March). In contrast, developmental timing in *Idesia* was found to be very different. No identifiable inflorescence meristems were found in buds sampled in February, although well-developed leaf primordia were present (**Figure 5**). Resampling in April revealed dramatic differences. A well-developed inflorescence meristem was found to be present, but no developed flowers (**Figure 5**). We conclude that inflorescence development in *Idesia* occurs in response to warming temperatures in the spring, although much of it is completed within the closed bud before bud break in May. At an early stage, the inflorescence meristem resembles a catkin in having numerous spirally arranged bracts and primordia on an axis. However, these primordia will develop into inflorescence branches and not individual flowers.

## Discussion

### Evolutionary-developmental Mechanisms Implicated in Inflorescence Evolution in the Salicoid Clade of Salicaceae

When the highly reduced and specialized inflorescence of *Salix* (**Figure 7**) is compared with its outgroup genera, for instance *Idesia* (**Table 3**), there are several traits that are shared. Our comparative investigation indicates that these apparently pre-existing shared traits (unisexual flowers, dioecy and association of flowering with resting buds) likely pre-date the evolution of catkins rather than being a consequence of the evolution of catkins. In contrast to the monoecious Fagales, where typically male and female catkins occur on the same tree, dioecy is universal in the catkin-bearing Salicaceae and their immediate relatives. Although bisexual teratomorphs are sometimes found, bisexual species in the catkin-bearing Salicaceae are exceedingly rare, although they do occur as a derived condition (Rohwer and Kubitzki, 1984). Dioecy in the group is ancient and stable and under genetic control (Geraldes et al., 2015), even though the genetic mechanism is labile and has apparently undergone numerous shifts within the genome (Filatov, 2015; Geraldes et al., 2015).

**Table 3:** Comparison of *Idesia* and *Salix* in terms of putative processes and characteristics of inflorescence evolution and development.

Process/characteristic	*Idesia*	*Salix*
Unisexual flowers	Yes	Yes
Dioecy	Yes	Yes
Flowering linked to resting buds	Yes	Yes
Preformation	No (or short)	Yes (long)
Contraction	No (elongated branched rachis)	Yes (complete)
Non-terminal deletion	No	Yes (in most species)
Precocity	No	Yes
Bud dimorphism	No (buds producing leaves and inflorescence)	Yes (buds either vegetative or flowering)
Floral reduction	No	Yes
Lateralization	No (inflorescence terminal)	Yes (inflorescence lateral)

A number of other traits, however, are specific to the catkin-bearing habit: preformation, precocity, bud dimorphism, inflorescence contraction, floral reduction, lateralization. These traits represent necessary steps to the evolution of catkins in the Salicaceae. They will be discussed in turn.

#### Preformation

This is the formation of structures a long time before they become visible or functional. In *Salix* and *Populus* the inflorescence is initiated as soon as the resting buds form, which may be as early as May in the year preceding flowering. The early initiation allows time for the catkin to be fully formed by the time buds break in the spring. By contrast, other members of the salicoid clade complete inflorescence maturation only after bud break in the spring (contrast **Figures 5A,B** with **Figures 6** and **7**). Inflorescence development may start in the bud (partial preformation) but completes on the growing shoot (see results and **Figure 5**). Preformation is obviously a necessary precondition for precocity (below), and precocity may be what is driving full preformation. Preformation in resting buds involves a paradox. In a normal bud there is no leaf production and shoot growth has ceased. The bud is in developmental stasis, at least vegetatively, in preparation for full dormancy. On the other hand with floral preformation there is much reproductive development in the bud with the formation of an inflorescence meristem and floral primordia and their development into flowers. The evolution of preformation implies increased developmental control in coupling two phase changes of the meristem: change from active growth to dormancy and change from vegetative to reproductive. In *Idesia* these phase changes seem to occur sequentially, first dormancy then inflorescence formation. In poplar and willow they appear to be coupled.

#### Precocity

The term precocity is here applied to the breaking of reproductive buds before the vegetative buds to allow flowering before the development of a canopy. It should be noted that precocity as discussed here is seasonal precocity, not flowering as a juvenile (also sometimes refers to as precocious flowering). Seasonal precocity implies bud dimorphism (below). Although most willows are strictly precocious, the genus *Salix* as a whole shows a great deal of variation in this trait. Some are what salicologists call “coetaneous” (an archaic word for contemporaneous), meaning that the catkins are produced at the same time (contemporaneously) with the leaves. More precisely this means that the catkins are not sessile on the previous years growth but on, bud-break, a leafy shoot is produced which the catkin terminates. In this respect it is directly analogous with *Idesia*, in which the inflorescence terminates a short leafy shoot. A third type of reproductive behavior, “serotiny” (from the latin *serotinus* = coming late), also occurs in willows. This is an extreme form of the coetaneous habit. In botany serotiny is more commonly applied to delayed seed dispersal, but in willows it refers to delayed flowering. This occurs when catkins are poorly developed in bud and complete their development post-budbreak, thus apparently flowering with the current season’s growth. Although formal analyses are lacking (partly due to continuing uncertainty over the phylogeny of *Salix*), it is likely that coetaneous and serotinous willows are derived and may represent reversals. However, if the coetaneous habit is found to be primitive in *Salix* it provides a link to other genera of the salicoid clade.

#### Non-terminal Deletion

Closely associated with precocity, non-terminal deletion is the evolutionary loss of parts of an organ from the base rather than the tip. In this case, it refers to the loss of leaves below the terminal inflorescence, as in precocious *Salix* and *Populus*. For instance *Idesia* bears its inflorescences on leafy shoots whereas the catkins of *Populus* are not associated with vegetative leaves. Evolutionary loss at the end of a shoot may simply be the consequence of growth ceasing early, while evolutionary gain at the tip may be the result of growth continuing for a longer period. Loss (in this case of leaves) at the base of a shoot is more problematic. It requires that a late developmental program (in this case inflorescence production) is brought forward to replace early developmental programs (in this case leaf primordia production which would normally take place as the resting buds form). The concepts of terminal and non-terminal deletion have been used in evolutionary analyses of other botanical systems, including of fossils (Bateman, 1994). In our present system we can see that extreme inflorescence preformation, characteristic of *Populus* and *Salix*, brings forward inflorescence production to precisely the time when leaves would be forming during the development of the resting bud. Therefore precocity, preformation and non-terminal deletion, although separate concepts, may in fact be interlinked parts of a single evolutionary scenario.

#### Bud Dimorphism

In *Salix* and *Populus* there is a functional dimorphism between floral and vegetative buds. Precocity implies that floral and vegetative buds may have different temperature sensitivity, with inflorescence buds having a lower cumulative temperature requirement (heat sum, for instance in degree days) required for development. Bud dimorphism allows a marked “division of labor” between reproductive and vegetative meristems. In poplar and willow the catkin usually has no vegetative function whatsoever, and correspondingly the vegetative shoot has no reproductive function. In *Idesia* the distinction is blurred. Almost all shoots produce not only a terminal inflorescence but also numerous leaves below the inflorescence. Thus reproductive and vegetative functions are carried out by the same buds (repro-vegetative buds).

#### Contraction

In most genera of the salicoid clade the inflorescence is a lax branched panicle with elongated rachises. The evolution of the catkin therefore requires evolutionary and developmental contraction of the inflorescence. The inflorescence meristem of *Idesia*, with primordia and associated bract primordia (**Figure 5**) gives a possible scenario of how the catkin could have evolved. The primordia would normally develop into panicle branches and then into flowers. If the floral developmental pathway were to be brought forward in developmental time then it is possible to see how the result would be a series of bract associated flowers. This would be an example of heterochrony: a change in developmental timing (Bateman, 1994; Rudall and Bateman, 2004). In Solanaceae, it is suggested that minor changes in the maturation process of apical meristems can give rise to dramatic changes in reproductive shoot organization (Park et al., 2012, 2014). In grasses, more complex panicles can be formed by delaying the phase change from the indeterminate shoot meristem (SM) inflorescence building program to a determinate spikelet and floral meristem (FM) program (Kyozuka et al., 2014). In the evolution of catkins we propose the reverse: a simplification of the panicle by early phase change from an inflorescence building (SM) program to a determinate FM developmental program. It is of interest that Fisher (Fisher, 1928a,b) found, in the catkins of some species of *Salix*, microstructures that she interpreted as the vestigial branches of an ancestral branched inflorescence. This implies that catkin evolution proceded via a progressive shortening of axes rather than a complete deletion of the branching pathway for inflorescence development. Furthermore, the floral developmental pathway has not been brought forward so far as to eliminate all trace of branch structure. Fisher’s finding was all the more remarkable as it came long before the appropriate outgroups were known and it was easy to assume that the ancestral form was a simple raceme rather than a branched panicle.

#### Floral Reduction

Small flowers are an obvious consequence of the evolution of the catkin as there is no space for elaborate flowers in a highly condensed inflorescence. Additionally some of the functions of individual flowers are, in the catkin, taken over by the inflorescence as a whole. An example is floral protection in bud which is done by the calyx in *Idesia* but by the tight packing of the flowers and investing bracts in poplar and willow. A remaining question is whether the calyx has been lost completely or converted into disk glands (nectaries) in willow. For nearly a century this question has been considered closed with the consensus that the disk glands of willow and the cupular disk of poplar represent the lost perianth. However, the recent identification of the relatives of *Salix* and *Populus* followed by the realization that they have both disk glands and a calyx have cast some doubt on this consensus (Alford, 2005). In addition to the loss of calyx there has also been a reduction in stamens in the insect-pollinated *Salix* (down to one in some species). In the wind-pollinated *Populus*, large numbers of stamens have been retained, packed very tightly into the flowers in bud. This illustrates the constraint that the more pollen-wasteful process of wind pollination places on floral reduction in poplar.

#### Lateralization

In *Idesia*, the terminal buds on shoots produce inflorescences and the inflorescence terminates shoot growth. In poplar, terminal buds are never (or at least very rarely) reproductive. The catkin buds are all lateral (axillary) buds. The determinate growth of all terminal buds puts a constraint on the rate of height growth that can be attained by *Idesia* and its relatives which tend to be relatively small trees. Poplar, however, because its inflorescences are lateral, can maintain indeterminate growth, resulting in poplars being generally the fastest growing and tallest dicotyledonous trees in the northern hemisphere. Catkins are also lateral in willow, but in willow the terminal shoot tends to abort rather than form a resting bud for continued growth the following year, hence willows also tend to be smaller in stature. In the Juglandaceae, lateralization of the catkins is only partial, as while staminate catkins are general lateral, pistillate catkins are usually terminal (Manning, 1938). This could suggest a physiological constraint between investment in large fruit (as in Juglandaceae) and inflorescence position.

### Adaptive Significance of Inflorescence Evolution in Salicaceae

The compactness of the catkin allows inflorescence development to be completed within the inflorescence bud. This in turn allows for precocious flowering. Precocity has an obvious consequence for wind-pollinated plants (such as poplars, *Populus*) as it allows flowers to be pollinated before the emergence of the leafy canopy which may attenuate air movement among the branches. For insect-pollinated plants, precocity effectively removes competition for bees from other flowers. Willows (*Salix*) are generally insect pollinated (Karrenberg et al., 2002), particularly by bees of the genus *Andrena* (Knuth, 1909; Ostaff et al., 2015). They are also well known to be an important source of pollen and nectar for honey bees (*Apis mellifera* L.) early in the year when bees have few other food sources. However, a trade-off against the absence of competition for pollinators, is the fact that there may be fewer bees flying in early months of the year. This mechanism, of course, applies only to temperate regions with a pronounced cold season, and is not applicable to catkin-like inflorescences of tropical origin such as the related Lacistemataceae.

Another mechanism, related to precocity, is thermal protection (Tsukaya and Tsuge, 2001). The contraction of the flowers into a compact inflorescence allows the flowers to be protected by hairs on the margins of the bracts. These form, in some instances, a striking wooly insulating layer around the catkin. Indeed, the name ‘catkin’ alludes to this flocculence. The woolliness is equivalent to the wooly hairs of many alpine plants and by trapping air may allow flowers to survive the severe night frosts encountered as a consequence of precocity. A lax panicle, on the other hand, cannot be protected by hairs on its bracts.

A third mechanism that should be considered is reproductive efficiency. Poplars and willows produce large amounts of seed with little investment in inflorescence structures. Compare this with *Idesia*, which produces a modest amount of seed with a heavy investment in inflorescence rachis and flower stalks. Furthermore, individual flowers of *Idesia* are large. They have to be, as each one has to provide a sufficient landing surface for pollinating insects. By aggregating minute flowers together, *Salix* provides a landing platform for bees while minimizing investment in individual flowers. This is an example of synorganization, i.e., the provision of a novel or more efficient function by different plant organs working in concert, in this case numerous small flowers organized into a larger unit that can function as a landing surface.

### The Genome-enabled Family Salicaceae as a Promising System for Evolutionary Developmental Biology

The salicoid clade of the Salicaceae exhibits a promising range of ecologically important morphological traits (Cronk, 2005). It is also one of the best characterized clades of dicotyledons at the genome level. The poplar (*P. trichocarpa* Torr. & A.Gray) genome was the third plant genome to be released (Tuskan et al., 2006) and it has now been joined on the comparative genomics site Phytozome (Goodstein et al., 2012) by the genome of *Salix purpurea* L., an economically important basket and biofuel willow extensively used in breeding programs for crossing with other species. Complete genome sequencing projects are well advanced for other species of *Salix* and *Populus* and a plethora of genomic information will soon be available. This raises the possibility of a molecular approach to the evolution of many key traits in the salicoid clade, including inflorescence architecture. Importantly for reproductive traits, the genomic architecture of the sex locus in *P. trichocarpa* has recently been elucidated (Geraldes et al., 2015).

Inflorescence architecture is a economically important trait in many crop species. The grapevine (*Vitis)* is a good example, in which a compact or lax infructescence (caused by variation of rachis length) is a characteristic of commercial importance (Correa et al., 2014). For obvious reasons the genes underlying this trait are now attracting increased attention. A number of mutants are known in *Arabidopsis* that affect inflorescence traits. An example of genes of potential relevance to catkins are the compact inflorescence (CFL) genes (Goosey and Sharrock, 2001).

The rich genomic resources developing for *Salix* and *Populus* will greatly facilitate the development of genomic resources for other genera in the salicoid clade. A complete genome of *Idesia* would be particularly valuable as an outgroup for *Salix* and *Populus*. Similarly, a member of the Salicaceae that is more distant (such as *Azara*) would be useful as an outgroup for the salicoid clade as a whole. *Azara* is outside the palaeopolyploidy event that has occurred in the salicoid clade, it would therefore be particularly useful to assess evolution of gene paralogues in *Salix* and *Populus* resulting from the whole genome duplication event.

## Conclusion

The morphological richness of the Salicaceae coupled with the rapidly expanding genomic resources make this family, of all woody plant families, particularly promising for genome-enabled evolutionary developmental biology.

## Author Contributions

QC and PR planned the study, collected material, supervised the anatomical work and wrote the paper. IN carried out the anatomical work and photomicrography and contributed to writing the paper.

## Conflict of Interest Statement

The authors declare that the research was conducted in the absence of any commercial or financial relationships that could be construed as a potential conflict of interest.
